# Metabolism and biochemical properties of nicotinamide adenine dinucleotide (NAD) analogs, nicotinamide guanine dinucleotide (NGD) and nicotinamide hypoxanthine dinucleotide (NHD)

**DOI:** 10.1038/s41598-019-49547-6

**Published:** 2019-09-11

**Authors:** Keisuke Yaku, Keisuke Okabe, Maryam Gulshan, Kiyoshi Takatsu, Hiroshi Okamoto, Takashi Nakagawa

**Affiliations:** 1 Department of Metabolism and Nutrition, Graduate School of Medicine and Pharmaceutical Science for Research, University of Toyama, Toyama, 930-0194 Japan; 2 First Department of Internal Medicine, Graduate School of Medicine and Pharmaceutical Science for Research, University of Toyama, Toyama, 930-0194 Japan; 3 Toyama Prefectural Institute for Pharmaceutical Research, Toyama, 939-0363 Japan; 4 Department of Immunobiology and Pharmacological Genetics, Graduate School of Medicine and Pharmaceutical Science for Research, University of Toyama, Toyama, 930-0194 Japan; 5 Department of Biochemistry, Tohoku University Graduate School of Medicine, Sendai, 980-8575 Japan; 6 Department of Biochemistry and Molecular Vascular Biology, Kanazawa University Graduate School of Medical Sciences, Kanazawa, 920-8640 Japan; 7 Institute of Natural Medicine, University of Toyama, Toyama, 930-0194 Japan

**Keywords:** Enzymes, Endocrine system and metabolic diseases

## Abstract

Nicotinamide adenine dinucleotide (NAD) is an important coenzyme that regulates various metabolic pathways, including glycolysis, β-oxidation, and oxidative phosphorylation. Additionally, NAD serves as a substrate for poly(ADP-ribose) polymerase (PARP), sirtuin, and NAD glycohydrolase, and it regulates DNA repair, gene expression, energy metabolism, and stress responses. Many studies have demonstrated that NAD metabolism is deeply involved in aging and aging-related diseases. Previously, we demonstrated that nicotinamide guanine dinucleotide (NGD) and nicotinamide hypoxanthine dinucleotide (NHD), which are analogs of NAD, are significantly increased in Nmnat3-overexpressing mice. However, there is insufficient knowledge about NGD and NHD *in vivo*. In the present study, we aimed to investigate the metabolism and biochemical properties of these NAD analogs. We demonstrated that endogenous NGD and NHD were found in various murine tissues, and their synthesis and degradation partially rely on Nmnat3 and CD38. We have also shown that NGD and NHD serve as coenzymes for alcohol dehydrogenase (ADH) *in vitro*, although their affinity is much lower than that of NAD. On the other hand, NGD and NHD cannot be used as substrates for SIRT1, SIRT3, and PARP1. These results reveal the basic metabolism of NGD and NHD and also highlight their biological function as coenzymes.

## Introduction

Nicotinamide adenine dinucleotide (NAD) is an essential cofactor that mediates various redox reactions through the transfer of electrons between NAD+ (oxidized form of NAD, hereafter referred to as NAD) and NADH (reduced form of NAD, hereafter referred to as NADH). NAD also serves as a substrate for poly(ADP-ribose) polymerase (PARP), sirtuin, and NAD glycohydrolase (CD38 and CD157)^1^. Although NAD is a well-studied metabolite that was discovered more than one hundred years ago^2^, it has been in spotlight recently due to its anti-aging effects^3–5^. Many studies have demonstrated that NAD levels decrease with aging in various tissues of rodents and humans, and the decline of NAD levels is involved in the pathogenesis of aging-related diseases, such as obesity, diabetes, and Alzheimer’s disease^3–5^. Furthermore, Supplementation of NAD precursors, such as nicotinamide mononucleotide (NMN) and nicotinamide riboside (NR), restores the decline of NAD levels with aging and ameliorates aging-associated physical declines and disease phenotypes^6–10^. In mammals, NAD is synthesized via *de novo*, Preiss-Handler, and salvage pathways from tryptophan, nicotinic acid, and nicotinamide (NAM), respectively^11^. In particular, the salvage pathway is the most important for the maintenance of NAD levels in mammals, and nicotinamide phosphoribosyltransferase (Nampt) is a rate-limiting enzyme in this pathway^12^. Nampt converts NAM and phosphoribosyl pyrophosphate (PRPP) to NMN^12^. Then, nicotinamide mononucleotide adenylyltransferase (Nmnat) transfers an adenylyl moiety from ATP to NMN to generate NAD^13^. In mammals, there are three Nmnat isozymes (Nmnat1–3) with different subcellular localizations and tissue distributions. Nmnat1, Nmnat2, and Nmnat3 have been considered to be in the nucleus, Golgi apparatus, and mitochondria, respectively^14^. However, we have demonstrated that Nmnat3-deficient mice have normal mitochondrial NAD levels compared with those in wild-type mice^15,16^. Moreover, overexpression of Nmnat3 in mice efficiently increases mitochondrial NAD levels with favorable effects on metabolic decline with aging^17^. These results indicate that Nmnat3 is dispensable for mitochondrial NAD maintenance, but overexpression of Nmnat3 in mice is sufficient to increase mitochondrial NAD levels. Interestingly, we have also found that the NAD analogs, oxidized forms of nicotinamide guanine dinucleotide (NGD+, hereafter referred to as NGD) and nicotinamide hypoxanthine dinucleotide (NHD+, hereafter referred to as NHD), are significantly increased in Nmnat3-overexpressing (Nmnat3 Tg) mice^17^. Previous reports have demonstrated that recombinant human and murine Nmnat proteins have enzymatic activities to generate NGD or NHD with guanosine triphosphate (GTP) or inosine triphosphate (ITP) instead of ATP^14,18^. Among the three Nmnat isozymes, Nmnat3 has a higher activity toward NGD and NHD compared to that of Nmnat1 and Nmnat2^14,18^. However, there is insufficient knowledge about the metabolism and functions of NGD and NHD *in vivo*. In the present study, we aimed to investigate the metabolism and biochemical properties of NGD and NHD.

## Results

### Measurement of NAD, NGD, and NHD by Orbitrap LC/MS

**Figure 1:**
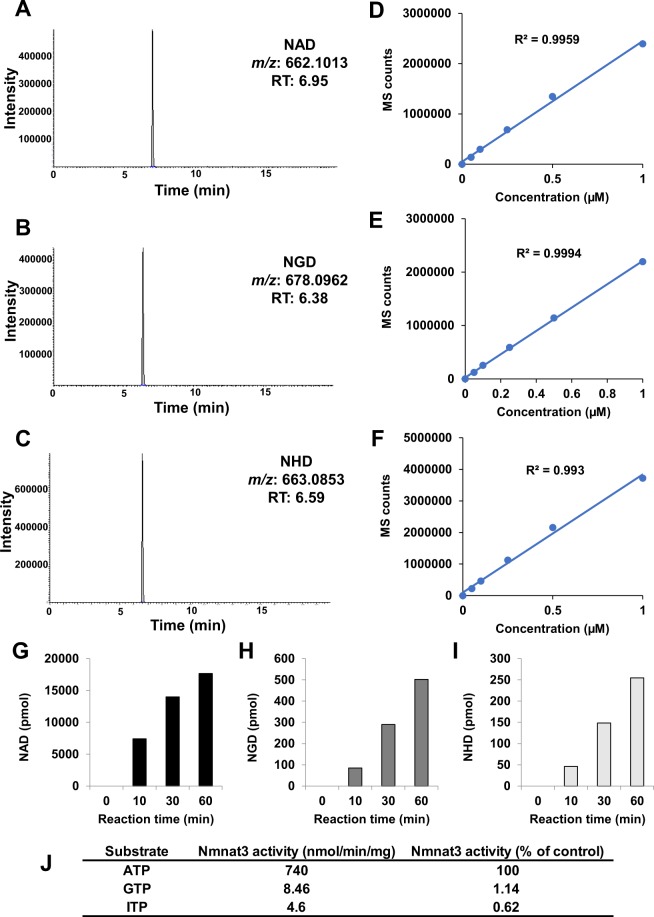
Detection of NAD analogs by using Orbitrap LC/MS system. (**A**–**F**) Representative chromatograms of standard compounds and standard curves of NAD (**A**,**D**), NGD (**B**,**E**), and NHD (**C**,**F**). Standard curves were calculated by measuring the known standard solution. X-axis represented concentrations of each compounds, and Y-axis represented the integrated sum of peak area from each chromatograms. (**G**–**I**) Recombinant human Nmnat3 produced NAD (**G**), NGD (**H**), and NHD (**I**) in *in vitro* reaction. (**J**) Production rate of NAD analogs by Nmnat3. The relative specificities against ATP were calculated from each production rate.

NAD is an abundant metabolite, and the quantification method has been established^19–21^. However, the amounts of NGD and NHD in cells are assumed to be extremely low. The difference in monoisotopic mass between NAD (MW 663.1091) and NHD (MW 664.0931) is less than 1 Da, and their chemical structures are quite similar. Therefore, the triple quadrupole mass spectrometer cannot distinguish between NHD and isotopomers of NAD. To distinguish between these NAD analogs, we employed an accurate, high-resolution mass spectrometer, Orbitrap, which can discriminate between *m/z* with less than 3 ppm accuracy. Standard samples of NAD, NGD, and NHD have similar retention times on high-performance liquid chromatography (HPLC) separation, but we specifically distinguished these NAD analogs using Orbitrap with a scan mode (Fig. 1A–C). We also confirmed that a linear regression was obtained between the concentrations of standards and mass spectrometer counts in each NAD analog (Fig. 1D–F).

### Nmnat3 synthesizes NGD and NHD from GTP and ITP *in vitro*

It has been previously reported that human and murine recombinant Nmnat3 proteins have enzymatic activities that generate NGD and NHD^14,18^. To confirm this, we assayed the enzymatic activities of Nmnat3 *in vitro* using our method. A recombinant human Nmnat3 protein was incubated with NMN and ATP, GTP, or ITP, and the production of NAD, NGD, and NHD was quantified using Orbitrap. We confirmed that Nmnat3 exactly catalyzed the production of NGD and NHD *in vitro* (Fig. 1G–I). However, the production rates of NGD and NHD were much lower than those of NAD (Fig. 1J).

### Physiological levels of NAD, NGD, and NHD in various murine tissues

**Figure 2:**
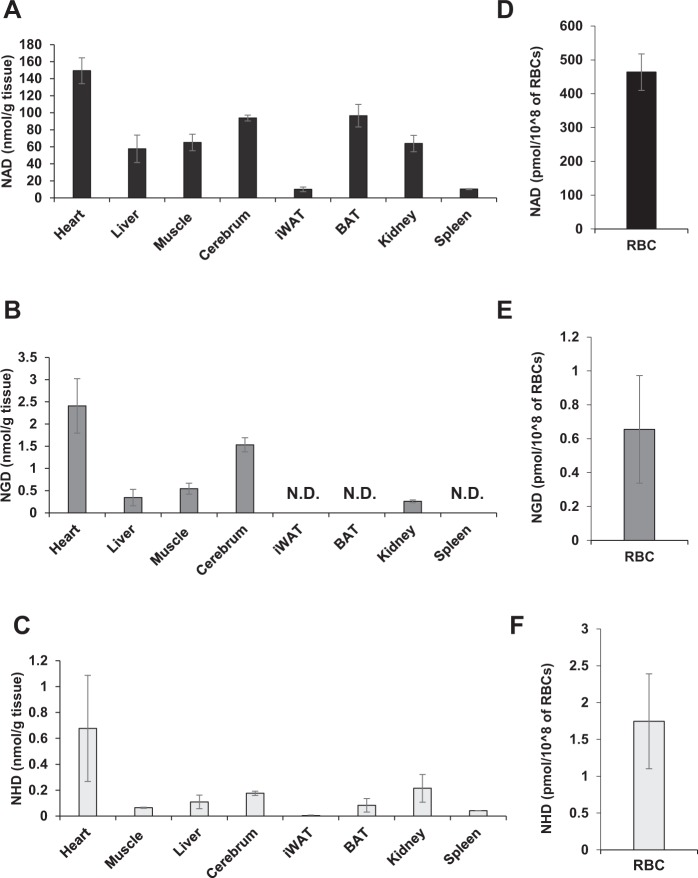
Quantification of NAD analogs in animal tissues. (**A**–**F**) Levels of NAD analogs, NAD (**A**,**D**), NGD (**B**,**E**), and NHD (**C**,**F**), in murine tissues, including heart, liver, skeletal muscle, cerebrum, inguinal white adipose tissue (iWAT), brown adipose tissue (BAT), kidney, spleen and RBCs from WT mice. Data are presented as means ± SD (n = 4).

NAD is ubiquitous in human and rodent tissues. However, the endogenous concentrations of NGD and NHD *in vivo* are not known yet. To evaluate the physiological levels of NGD and NHD, we measured their levels in several murine tissues using Orbitrap (Fig. 2A–C), and we detected them in most of the tissues we tested. In particular, the heart contained the largest amount of NGD and NHD (Fig. 2B,C). Although NGD was more abundant than NHD in all of the tissues, their levels were less than 3% of NAD level. We also investigated the levels of NGD and NHD in red blood cells (RBCs), where Nmnat3 is a dominant Nmnat isozyme^16^, and detected considerable amounts of both (Fig. 2D–F). In summary, NGD and NHD exist in various murine tissues; however, their levels are much lower than that of NAD.

### Levels of NAD, NGD, and NHD during aging

Many studies have reported that NAD levels decline with aging in multiple tissues^6,21–24^. It has been demonstrated that decreased NAD synthesis and increased degradation of NAD contribute to the decline in its levels with aging^4,5^. In particular, expression of Nampt significantly decreases during aging^6,25,26^. Because NGD and NHD are also generated from NMN, a product of Nampt, we assume that their levels of NGD and NHD are also affected by aging. In agreement with previous reports, NAD levels in skeletal muscle and the liver were significantly reduced in aged mice (Table 1). Of note, we found that NGD levels in heart and skeletal muscle significantly decreased with aging (Table 1). However, there were no significant differences in levels of NHD between young and aged tissues (Table 1). Thus, it is speculated that the regulation of levels of NAD analogs may differ among tissues.

**Table 1:** Levels of NAD, NGD, and NHD in young and aged mice.

		Young	Old	
NAD (nmol/g tissue)	Heart	170 ± 50	190 ± 30	NS
	Liver	79 ± 14	51 ± 23	p < 0.05
	Muscle	140 ± 20	87 ± 27	p < 0.05
NGD (nmol/g tissue)	Heart	1.1 ± 0.1	0.72 ± 0.09	p < 0.05
	Liver	0.86 ± 0.15	0.60 ± 0.16	NS
	Muscle	0.61 ± 0.12	0.41 ± 0.02	p < 0.05
NHD (nmol/g tissue)	Heart	0.62 ± 0.18	0.60 ± 0.31	NS
	Liver	0.28 ± 0.17	0.40 ± 0.30	NS
	Muscle	0.26 ± 0.08	0.21 ± 0.13	NS

Levels of NAD analogs in tissue samples prepared from young (3-month-old) and old (24-month-old) WT mice were measured by orbitrap. Data are presented as means ± SD (n = 4). NS means not significant.

### Enzymes involved in the synthesis and degradation of NGD and NHD *in vivo*

**Figure 3:**
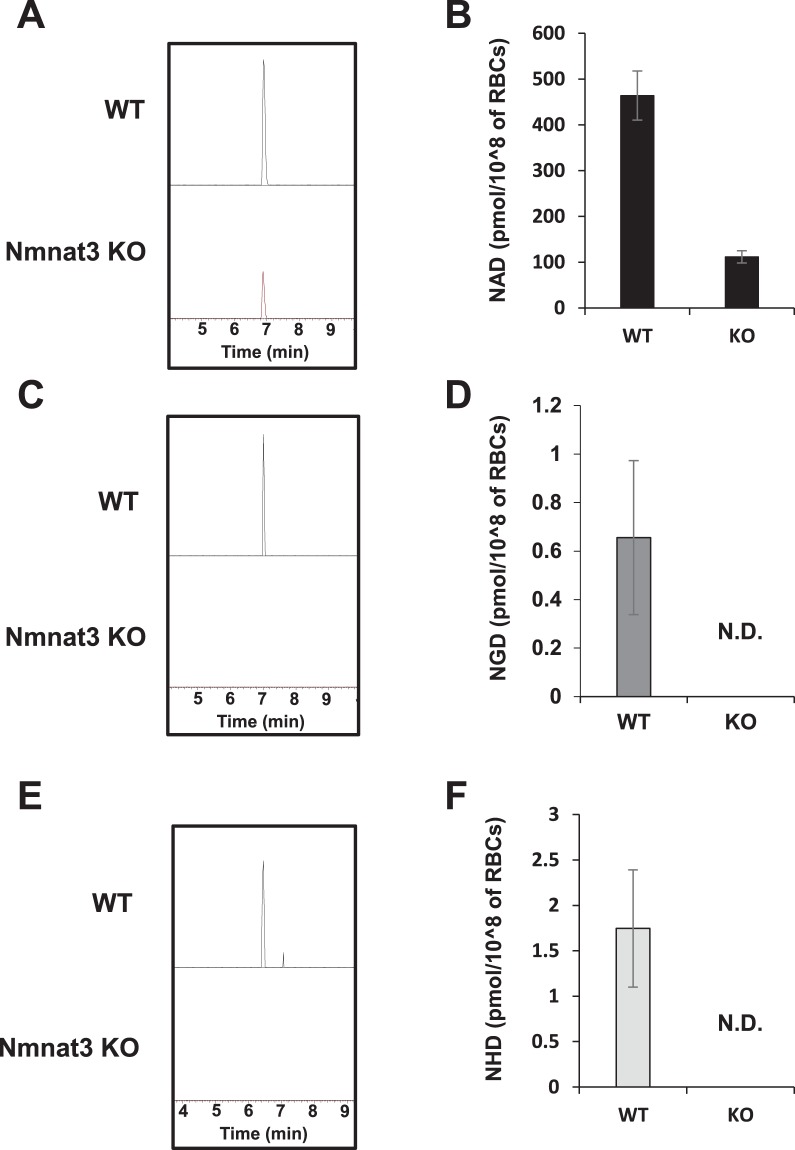
Nmnat3 contributes the production of NGD and NHD in RBCs. (**A**–**F**) Representative chromatograms and quantification of NAD (**A**,**B**), NGD (**C**,**D**), and NHD (**E**,**F**) in RBCs from WT and Nmnat3 KO mice. Data are presented as means ± SD (n = 4).

We previously reported that overexpression of Nmnat3 increased NAD, NGD, and NHD levels in skeletal muscle^17^. On the other hand, Nmnat3 deficiency decreased NAD levels in RBCs but not in other tissues^15,16^, and NGD and NHD levels in Nmnat3-deficient (Nmnat3 KO) mice are not known yet. To examine whether Nmnat3 is important for maintaining endogenous NGD and NHD levels, we measured their levels in Nmnat3 KO and Nmnat3-overexpressing (Nmnat3 Tg) mice. Consistent with our previous report, overexpression of Nmnat3 significantly increased NAD levels in heart and skeletal muscle but not in the liver (Table 2). Moreover, the levels of both NGD and NHD significantly increased in these tissues in Nmnat3 Tg mice (Table 2). The level of NGD in Nmnat3 Tg mice drastically increased to an extent comparable to that of NAD, but the increase in NHD level was slight. Further, we investigated NGD and NHD levels in Nmnat3 KO mice. We previously demonstrated that NAD levels were significantly reduced in Nmnat3 KO mice RBCs but were unchanged in other tissues^15,16^. Accordingly, NAD levels in RBCs were significantly reduced, and NGD and NHD were not detectable in RBCs of Nmnat3 KO mice (Fig. 3A–F). We also measured the levels of NAD analogs in other tissues and found that NGD levels in heart and skeletal muscle and NHD levels in heart were significantly reduced in Nmnat3 KO mice (Table 3). Furthermore, the levels of NGD and NHD in the liver of Nmnat3 KO mice were almost the same as those in wild-type (WT) controls (Table 3). These results indicate that the production of NGD and NHD in RBCs primarily depends on Nmnat3, but other Nmnat isozymes, such as Nmnat1, may also be involved in the production of NGD and NHD in other tissues, including the liver.

**Table 2:** Levels of NAD, NGD and NHD in Nmnat3 Tg mice.

		WT	Nmnat3 Tg	
NAD (nmol/g tissue)	Heart	150 ± 20	170 ± 4	p < 0.05
	Liver	58 ± 16	49 ± 10	NS
	Muscle	65 ± 10	95 ± 10	p < 0.05
NGD (nmol/g tissue)	Heart	3.1 ± 1.5	91 ± 18	p < 0.05
	Liver	0.34 ± 0.19	0.66 ± 0.16	NS
	Muscle	0.72 ± 0.46	130 ± 10	p < 0.05
NHD (nmol/g tissue)	Heart	0.68 ± 0.41	1.0 ± 0.4	NS
	Liver	0.14 ± 0.65	0.39 ± 0.20	NS
	Muscle	0.062 ± 0.002	0.39 ± 0.04	p < 0.05

Levels of NAD analogs in tissue samples prepared from 3-month-old Nmnat3 Tg and WT mice were measured by orbitrap. Data are presented as means ± SD (n = 4). NS means not significant.

**Table 3:** Levels of NAD, NGD and NHD in Nmnat3 KO mice.

		WT	Nmnat3 KO	
NAD (nmol/g tissue)	Heart	120 ± 10	98 ± 15	NS
	Liver	120 ± 40	130 ± 20	NS
	Muscle	190 ± 20	170 ± 20	NS
	Cerebrum	41 ± 12	58 ± 23	NS
NGD (nmol/g tissue)	Heart	2.2 ± 0.5	0.86 ± 0.13	p < 0.05
	Liver	1.5 ± 0.1	1.5 ± 0.2	NS
	Muscle	0.80 ± 0.12	0.59 ± 0.11	p < 0.05
	Cerebrum	0.66 ± 0.09	0.66 ± 0.11	NS
NHD (nmol/g tissue)	Heart	0.54 ± 0.03	0.83 ± 0.22	NS
	Liver	0.19 ± 0.05	0.18 ± 0.05	NS
	Muscle	0.21 ± 0.06	0.18 ± 0.04	NS
	Cerebrum	0.21 ± 0.04	0.21 ± 0.04	NS

Levels of NAD analogs in tissue samples prepared from 3-month-old Nmnat3 KO and WT mice were measured by orbitrap. Data are presented as means ± SD (n = 4). NS means not significant.

CD38 has been reported to be important for the regulation of NAD levels during aging^24,27^. CD38 is an ectoenzyme and exerts enzymatic activities, such as NAD glycohydrolase and ADP-ribosyl cyclase activities^28,29^. CD38 increases with age and consumes considerable amounts of NAD. In fact, CD38-deficiency in mice results in increased NAD levels and prevents aging-associated NAD decline in various tissues^24^. It has been reported that CD38 also exerts ribosyl cyclase activity toward NGD as well as NAD^30,31^. In this reaction, CD38 generates cyclic-GDP ribose (cGDPR) from NGD. Therefore, we measured the levels of NAD analogs in WT and CD38-deficient (CD38 KO) mice. As previously reported, NAD levels significantly increased in multiple tissues of CD38 KO mice (Table 4). On the other hand, NGD levels were increased only in the heart of CD38 KO mice. Unlike NAD levels, the levels of NGD and NHD in skeletal muscle were decreased in CD38 KO mice (Table 4).

**Table 4:** Levels of NAD, NGD and NHD in CD38 KO mice.

		WT	CD38 KO	
NAD (nmol/g tissue)	Heart	160 ± 20	460 ± 10	p < 0.05
	Liver	120 ± 40	450 ± 20	p < 0.05
	Muscle	190 ± 4	320 ± 30	p < 0.05
NGD (nmol/g tissue)	Heart	1.2 ± 0.1	1.8 ± 0.1	p < 0.05
	Liver	1.3 ± 0.3	1.6 ± 0.3	NS
	Muscle	0.74 ± 0.15	0.52 ± 0.09	p < 0.05
NHD (nmol/g tissue)	Heart	0.53 ± 0.15	0.61 ± 0.16	NS
	Liver	0.23 ± 0.16	0.23 ± 0.11	NS
	Muscle	0.16 ± 0.06	0.035 ± 0.04	p < 0.05

Levels of NAD analogs in tissue samples prepared from 3-month-old WT and CD38 KO mice were measured by orbitrap. Data are presented as means ± SD (n = 4). NS means not significant.

### Roles of NGD and NHD as coenzymes and substrates for sirtuin and PARP

**Figure 4:**
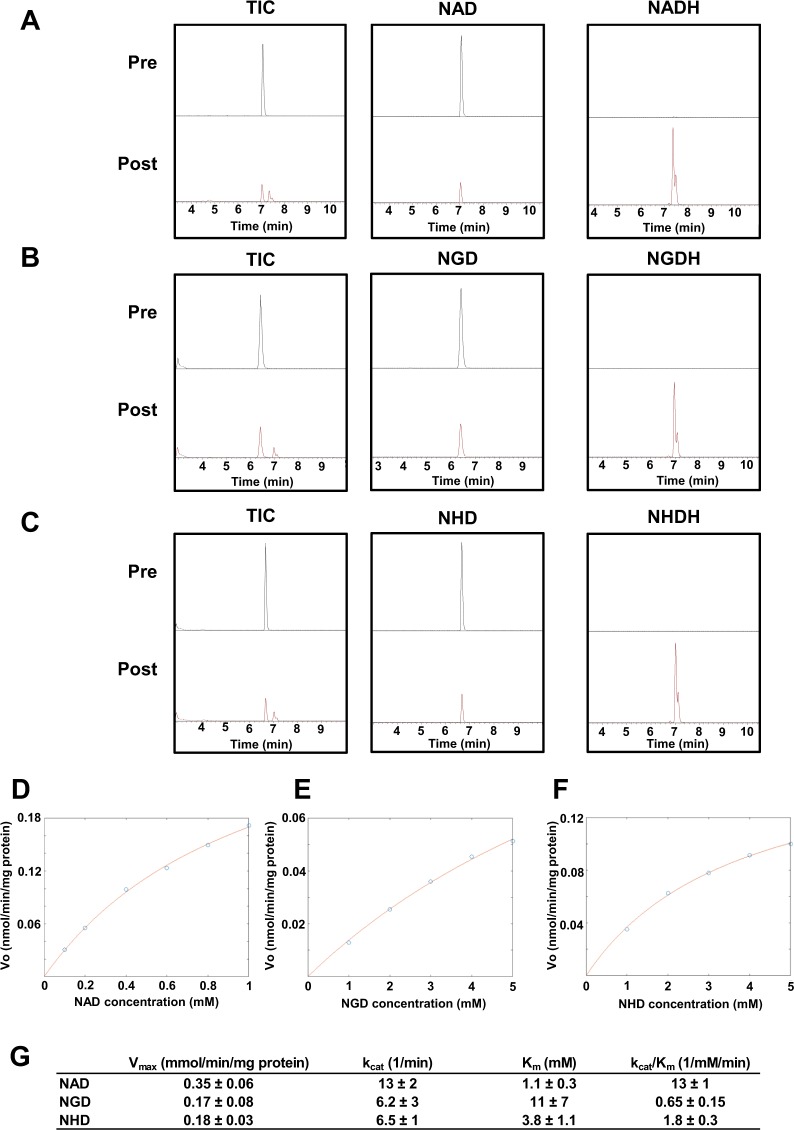
NGD and NHD act as coenzymes in ADH-mediated redox reaction. (**A**–**C**) Each NAD analog was incubated with ADH, and then pre- and post reaction samples were analyzed by orbitrap with a scan mode. Total ion chromatogram (TIC) and extracted chromatograms for oxidized and reduced forms of NAD (**A**), NGD (**B**), and NHD (**C**) are represented. (**E**–**G**) Non-linear fitting to Michaelis-Menten in ADH reaction incubated with various concentrations of NAD (**D**), NGD (**E**), and NHD (**F**), and apparent kinetic parameters, including V_max_, k_cat_, and K_m_, are calculated (**G**). The estimated parameters were calculated with 95% confidence intervals.

NAD is a coenzyme that mediates redox reactions through a transfer of electrons between NAD+ (its oxidized form) and NADH (its reduced form). Hundreds of enzymes use NAD as a coenzyme and regulate various metabolic pathways^32^. NGD and NHD are also believed to function as electron donors or acceptors. However, there is no direct experimental evidence for this function. Therefore, we investigated whether NGD and NHD function as coenzymes in a redox reaction. To investigate this question, we employed alcohol dehydrogenase (ADH), which catalyzes the oxidation of alcohol coupled with the reduction of NAD to NADH^33^. Using the reaction with ADH, we examined the formation of NGDH and NHDH from NGD and NHD, respectively. Analyses using Orbitrap revealed that NAD, NGD, and NHD peaks decreased after the reaction with ADH. Furthermore, peaks corresponding to NADH, NGDH, and NHDH could be detected after the reaction (Fig. 4A–C). We also confirmed that absorbance at 340 nm increased in correlation with NGDH and NHDH production (data not shown). These results suggest that NGD and NHD can mediate redox reactions as coenzymes. We also examined the substrate specificity of NAD analogs against ADH by measuring the apparent values of k_cat_ and K_m_ of NAD analogs with 150 mM ethanol (Fig. 4D–G). As expected, ADH had the highest specificity toward NAD as determined by k_cat_ and K_m_ values. In addition, NHD had a higher affinity for ADH than did NGD (Fig. 4G). These results indicate that NGD and NHD can serve as coenzymes but that they have an affinity for ADH that is much lower than that of NAD.

**Figure 5:**
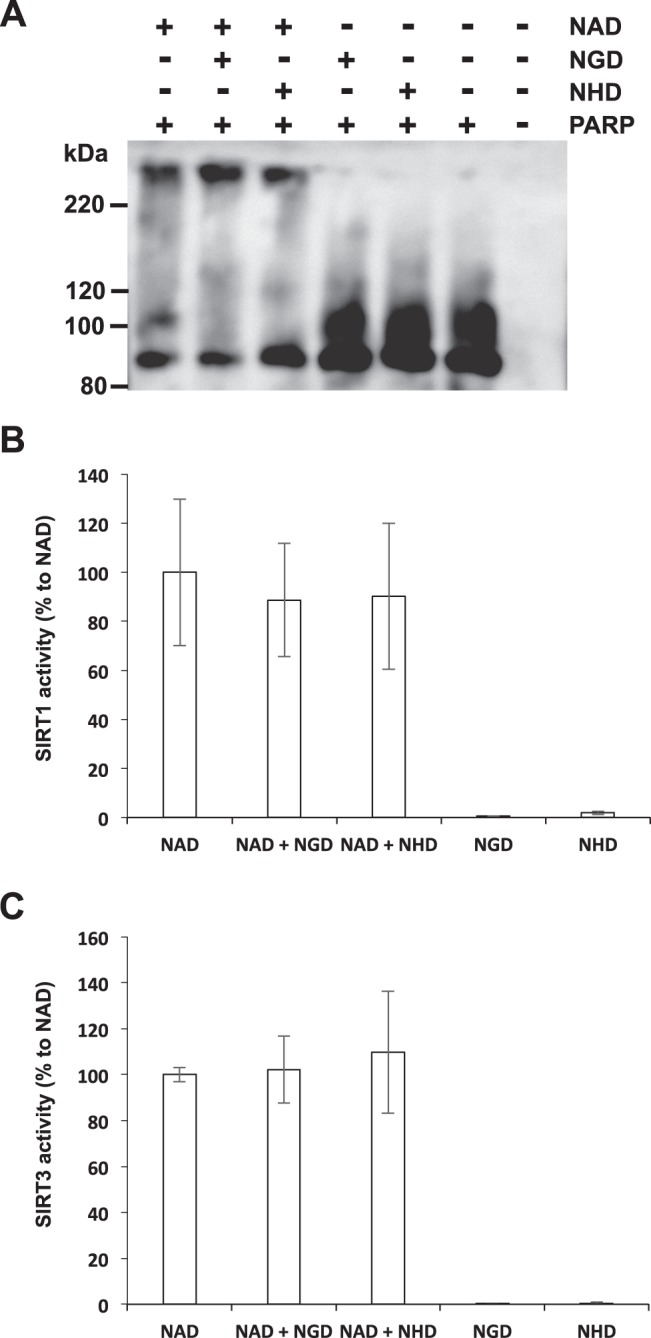
NGD and NHD cannot serve as substrates for PARP and sirtuins. (**A**) Immunoblot analysis of PARP1 activity. Auto-conjugation of each NAD analog was detected by Western blotting using anti-PARP1 antibody. (**B** and **C**) Deacetylaion activity was measured by using specific probes. Recombinant SIRT1 (**B**) and SIRT3 (**C**) was incubated with each NAD analog. Data are presented as means ± SD (n = 3).

NAD also serves as a substrate for poly(ADP-ribosyl)ation and deacetylation by PARPs and sirtuins, respectively^1^. Therefore, we investigated whether NGD and NHD served as substrates for PARP or sirtuin. First, we tested the effect of NGD and NHD on PARP activity *in vitro*. Recombinant PARP1 protein was incubated with NAD, NGD, or NHD, and then the conjugation of NAD analogs to PARP1 was visualized by Western blotting with anti-PARP1 antibody. As shown in Fig. 5A, the addition of NAD resulted in a smeared PARP1 band that represented the auto-poly-ADP-ribosylation of PARP1. However, addition of NGD and NHD had no effect on PARP1 bands. In addition, NGD and NHD had no inhibitory effect on PARP1-mediated auto-poly-ADP-ribosylation with NAD (Fig. 5A). We further investigated the effect of NAD analogs on sirtuin activity. Unlike NAD, both NGD and NHD exhibited no activity against SIRT1 and SIRT3 (Fig. 5B). We also examined whether NGD or NHD competitively inhibited sirtuin activity with NAD. We found that neither NAD analog had inhibitory effects against NAD-mediated SIRT1 or SIRT3 activities (Fig. 5B). We concluded that NGD and NHD could not serve as substrates for PARP or sirtuins.

## Discussion

Although previous studies have shown that Nmnat3 can generate NGD and NHD *in vitro*, there is little information about their existence *in vivo*. The present study demonstrates that NGD and NHD endogenously exist in various murine tissues and that their synthesis and degradation are partially mediated by Nmnat3 and CD38. We have previously demonstrated that a deficiency of Nmnat3 in mice reduces NAD revels in RBCs but not in other tissues. Similar to NAD, both NGD and NHD are completely absent in RBCs of Nmnat3 KO mice. These NAD analogs are also significantly reduced in other tissues where NAD levels are not affected. Previous *in vitro* studies have shown that murine Nmnat1 also exerts activities toward NGD and NHD as well as NAD^14,18^. These results suggest that Nmnat1 is also responsible for the generation of NGD and NHD in certain murine tissues, such as the liver. Thus, analyses using a Nmnat1-tissue specific KO mouse may be required to reveal the synthesis of NGD and NHD *in vivo*. Experiments using CD38 KO mice have shown that CD38 contributes to the regulation of NGD and NHD levels in some murine tissues. CD38 has enzymatic activities, such as NAD glycohydrolase and ADP-ribosyl cyclase, which generate ADPR and cADPR, respectively^28^. A previous *in vitro* study demonstrated that CD38 can generate cGDPR from NGD^30,31^. Thus, it is of interest to determine whether levels of cGDPR are changed in CD38 KO mice.

We also demonstrated that NGD and NHD serve as coenzymes for ADH, although their affinities for ADH are much lower than that of NAD. In the present study, we only tested whether NGD and NHD functioned as coenzyme in a redox reaction with ADH; however NGD and NHD may function in other NAD-dependent redox enzymes with higher affinity. Additionally, the biological function of NAD analogs *in vivo* is still unknown. Previously, we demonstrated that Nmnat3 KO mice exhibit hemolytic anemia^16^. Although the amounts of NGD and NHD in Nmnat3 KO RBCs are reduced to undetectable levels, NAD is also significantly reduced. A reduction in NAD levels impairs the activity of GAPDH and subsequent glycolysis flow and results in lowered ATP production in Nmnat3 KO RBCs^16^. Thus, it is difficult to determine whether the deficiency of NGD and NHD in Nmnat3 KO RBCs contributes to hemolytic anemia observed in Nmnat3 KO mice. Nmnat3 Tg mice exhibit metabolically beneficial effects against aging- and diet-associated insulin resistance with increased levels of NAD, NGD, and NHD^17^. In particular, NGD levels in Nmnat3 Tg mice increased drastically to the match the levels of NAD. Similar to the case of Nmnat3 KO mice, it is difficult to distinguish the contributions of NGD and NHD from the contribution of NAD in the phenotype observed in Nmnat3 Tg mice. However, we have shown that increased NGD levels slightly inhibited mitochondrial complex I activity in Nmnat3 Tg mice and may reduce the generation of reactive oxygen species from complex I. A recent study has demonstrated that NHD inhibits NAD synthesis in *E. coli*^34^. On the other hand, it was reported that NAD and NGD exhibited a synergistic effect in ADH reaction^35^. Therefore, these NAD analogs may contribute to metabolism by supporting or inhibiting NAD-related reactions. NGD- or NHD-specific redox enzymes may also exist. It is important to discover NGD- or NHD-preferred enzymes and determine their functions in Nmnat3 KO and Tg mice.

A previous study using radioisotope-labeled NGD reported that PARP could use NGD as a substrate^36^. However, our results using Western blotting indicated that NGD and NHD cannot be used as substrates for PARP1. Although it is still unclear what caused the discrepancy regarding PARP activity with NGD and NHD, there is a possibility that other PARPs can use NGD and/or NHD as a substrate. We also tested whether NGD and NHD serves as substrates for SIRT1 and SIRT3, but our results demonstrated that they couldn’t use both NGD and NHD for their deacetylation reactions. Reportedly, SIRT1 and SIRT3 mainly exert deacetylase activity, and other sirtuins exert deacylase activity^37^. Further studies are warranted to clarify the roles of NGD and NHD in sirtuin and PARP regulation.

## Materials and Methods

### Reagents and chemicals

NAD was obtained from Nacalai Tesque (Japan). NGD and NHD were purchased from Sigma-Aldrich (USA). LC/MS grade ultrapure water and methanol were obtained from Wako Pure Chemical Industries, Ltd. (Japan).

### Animals

C57BL/6 N mice were obtained from Japan SLC, Inc (Japan). CD38 knockout mice were obtained from RIKEN BRC (Stock No. RBRC01462)^38^. Details of Nmnat3 KO and Tg mice were described elsewhere^16,39^. Mice were maintained under controlled temperature and standard light conditions (12:12 h light-dark cycle) and were allowed free access to water and food. All animal experiments were approved by the Animal Experiment Committee at University of Toyama and carried out in accordance with the Guidelines for the Care and Use of Laboratory Animals at University of Toyama, which were based on international policies.

### Metabolite extraction from tissues

The excised animal tissues were immediately frozen in liquid nitrogen and kept in −80 °C until use. The frozen tissues were weighed and immersed in 50% methanol/50% water at 30 mg/600 µl. The tissues were grinded by multi beads shocker (Yasui Kikai, Japan). After centrifugation, the supernatant was collected in a new tube. Subsequently, 600 µl of chloroform was added and the solution was mixed by vortex for another 10 seconds. The mixture was centrifuged at 13,000 × *g* for 10 minutes at 4 °C. The upper phase (aqueous phase) was collected into a new tube and the same procedure was repeated again. Finally, the aqueous phase was dried and reconstituted in LC/MS grade water followed by filtration using 0.45 μm Millex filter unit (Merck Millipore, USA).

### LC/MS analysis

All the metabolites were measured by the Thermo Scientific LTQ Orbitrap XL ETD mass spectrometer combined with Accela HPLC systems (Thermo Fisher Scientific, USA). For the measurement, 10 µl of samples was injected to the system. NAD metabolites were separated through Atlantis T3 Column (2.1 × 150 mm, particle size 3 µm, Waters) using mobile phase A (5 mM ammonium formate) and mobile phase B (methanol) with a flow rate of 150 µl/min and a column temperature at 40 °C. The programmed mobile phase gradient was as following: 0–10 min, 0–70% B; 10–15 min, 70% B; 15–20 min, 0% B. The Atlantis T3 column was equilibrated prior to first injection. All samples were measured by FT-MS scan mode with negative ESI. To quantify NAD analogs, acquired data were extracted by mass range between the rigorous mass of NAD analogues up to plus and minus 0.01 and calculated by integrated sum of area using Xcalibur sofrware (Thermo Fisher Scientific, USA).

### Enzymatic activity assay of Nmnat3

To measure Nmnat3 activity, 1 µg of human recombinant Nmnat3 was incubated with 1 mM ATP, GTP, or ITP in 100 µL of reaction buffer (25 mM Tris-HCl, pH 7.4, 0.05 mM MgCl_2_, and 1 mM NMN) at room temperature. The reaction was stopped by adding 200 µL of 0.5 N perchloric acid. After centrifugation, the supernatant was neutralized by adding the same volume of 1 M ammonium formate followed by filtration using a 0.45 μm Millex filter unit (Merck Millipore, USA). The filtrated samples were analyzed by LC/MS as described above.

### Reduction of NAD analogs and measurement of ADH activity

To generate reduced form of NAD, NGD, and NHD, 1 mM of each NAD analog was incubated with ADH in the reaction buffer (20 mM Tris-HCl, pH 8.8, and 150 mM ethanol) for 2 hours at room temperature. The reaction was stopped with 0.5 N perchloric acid, and then the solution was neutralized with 1 M ammonium formate. Generation of NADH, NGDH and NHDH were confirmed by Orbitrap.

ADH activity was measured by monitoring the reduction of NAD, NGD and NHD using 340 nm absorbance. Various concentrations of NAD, NGD, and NHD was incubated with ADH with 150 mM ethanol in 20 mM Tris-HCl, pH 8.8 reaction buffer. The increase in absorbance of 340 nm was monitored by Varioskan Flash (Thermo Fisher Scientific, USA). The velocity was calculated based on molar absorbance coefficient obtained from NADH standard compound (6220 M^−1^cm^−1^), and the same molar absorbance coefficient was used for NGDH and NHDH based on previous reports^40–42^. Apparent values of V_max_ and K_m_ were calculated by using the non-linearization method (nlin function) in the MATLAB) to fit the Michaelis-Menten equation.

### PARP activity assay

To detect PAPR1 activity, 30 ng of recombinant human PARP1 (Biovision, USA) was incubated with 1 µg of sonicated salmon sperm DNA (BioDynamics Laboratory Inc. Japan) and 10 mM NAD, NGD, or NHD in 30 µL of reaction buffer (20 mM Tris-HCl, pH7.4, and 10 mM MgCl_2_) for 10 minutes. The reaction was terminated by adding SDS-PAGE sample buffer followed by boiling for 5 minutes at 95 °C. The samples were subjected to Western blotting with anti-PARP1 N-terminal antibody (Active Motif, USA). Chemiluminescent signals were detected by LAS 4000 Mini digital imager (GE healthcare, USA).

### Sirtuin activity assay

Sirtuin activities were measured by using SIRT1/Sir2 Deacetylase Fluorometric Assay Kit Ver.2 (MBL, Japan). Recombinant SIRT1 and SIRT3 were incubated with fluorescent substrate as described in the instruction, and the fluorescence intensity was monitored with excitation at 350 nm and emission at 450 nm by Varioskan Flash. (Thermo Fisher Scientific, USA).

### Statistical analysis

The significant differences were evaluated by using unpaired Student’s t-test. P-values less than 0.05 were determined as statistically significant. Data were expressed means ± SD.

## Supplementary information

Supplementary Information
