# Supplementary material for: Metabolism and biochemical properties of nicotinamide adenine dinucleotide (NAD) analogs, nicotinamide guanine dinucleotide (NGD) and nicotinamide hypoxanthine dinucleotide (NHD)
Source: Sci Rep. 2019 Sep 11;9:13102. doi: 10.1038/s41598-019-49547-6 (PMC6739475; doi:10.1038/s41598-019-49547-6)
Supplement: Supplementary file 1 — Supplementary Information [file 41598_2019_49547_MOESM1_ESM.pdf]

Supplementary Information file

Metabolism and biochemical properties of nicotinamide adenine dinucleotide (NAD) analogs, nicotinamide guanine dinucleotide (NGD) and nicotinamide hypoxanthine dinucleotide (NHD)

Keisuke Yaku[1], Keisuke Okabe[1,2], Maryam Gulshan[1], Kiyoshi Takatsu[3,4], Hiroshi Okamoto[5,6], Takashi Nakagawa[1,7]

[1]Department of Metabolism and Nutrition, Graduate School of Medicine and Pharmaceutical Science for Research, University of Toyama, Toyama 930-0194, Japan
[2]First Department of Internal Medicine, Graduate School of Medicine and Pharmaceutical Science for Research, University of Toyama, Toyama 930-0194, Japan
[3]Toyama Prefectural Institute for Pharmaceutical Research, Toyama 939-0363, Japan
[4]Department of Immunobiology and Pharmacological Genetics, Graduate School of Medicine and Pharmaceutical Science for Research, University of Toyama, Toyama 930-0194, Japan
[5]Department of Biochemistry, Tohoku University Graduate School of Medicine, Sendai 980-8575, Japan
[6]Department of Biochemistry and Molecular Vascular Biology, Kanazawa University Graduate School of Medical Sciences, Kanazawa 920-8640, Japan
[7]Institute of Natural Medicine, University of Toyama, Toyama 930-0194, Japan

Supplementary Figure 1

A

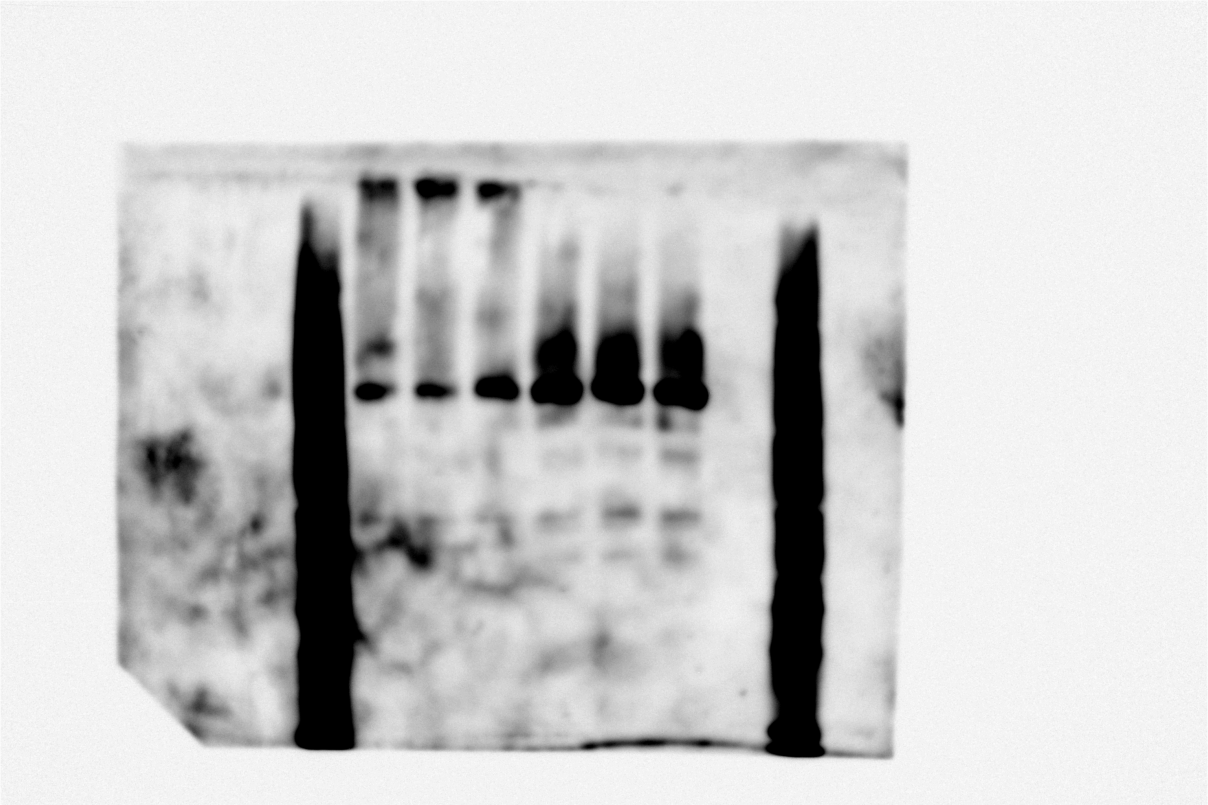

B

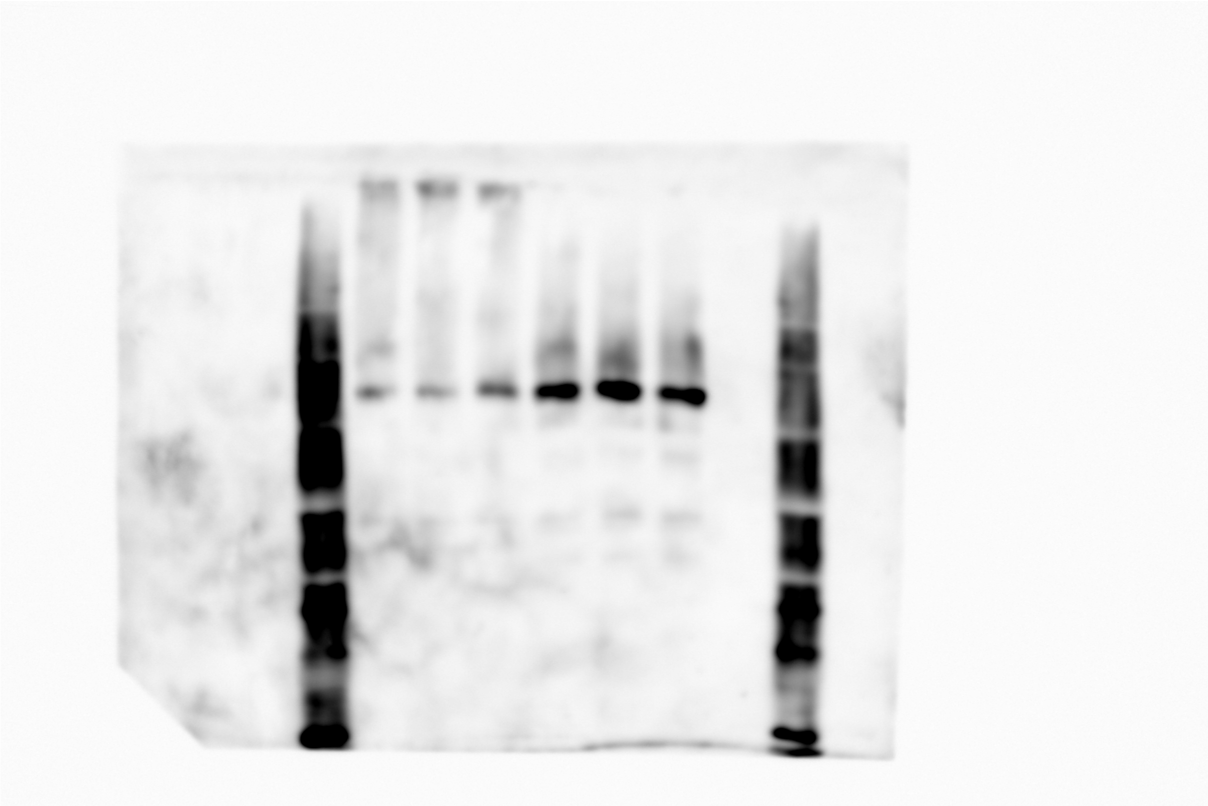

Supplementary Figure Legend

Supplementary Figure 1.

(A) Original gel image corresponded to Figure 5A. (B) Shorter exposure gel image corresponded to Figure 5A.
